# Intramembrane protease SPP defines a cholesterol-regulated abundance control of the mevalonate pathway enzyme squalene synthase

**DOI:** 10.1016/j.jbc.2024.105644

**Published:** 2024-01-11

**Authors:** Dönem Avci, Ronny Heidasch, Martina Costa, Christian Lüchtenborg, Dipali Kale, Britta Brügger, Marius K. Lemberg

**Affiliations:** 1 Center for Molecular Biology of Heidelberg University (ZMBH), DKFZ-ZMBH Alliance, Heidelberg, Germany; 2 Center for Biochemistry, Medical Faculty, University of Cologne, Cologne, Germany; 3 Biochemistry Center of Heidelberg University (BZH), Heidelberg, Germany

**Keywords:** intramembrane proteolysis, ER-associated degradation, mevalonate pathway, cholesterol, metabolic regulation

## Abstract

Intramembrane proteolysis regulates important processes such as signaling and transcriptional and posttranslational abundance control of proteins with key functions in metabolic pathways. This includes transcriptional control of mevalonate pathway genes, thereby ensuring balanced biosynthesis of cholesterol and other isoprenoids. Our work shows that, at high cholesterol levels, signal peptide peptidase (SPP) cleaves squalene synthase (SQS), an enzyme that defines the branching point for allocation of isoprenoids to the sterol and nonsterol arms of the mevalonate pathway. This intramembrane cleavage releases SQS from the membrane and targets it for proteasomal degradation. Regulation of this mechanism is achieved by the E3 ubiquitin ligase TRC8 that, in addition to ubiquitinating SQS in response to cholesterol levels, acts as an allosteric activator of SPP-catalyzed intramembrane cleavage of SQS. Cellular cholesterol levels increase in the absence of SPP activity. We infer from these results that, SPP-TRC8 mediated abundance control of SQS acts as a regulation step within the mevalonate pathway.

Cellular levels of key metabolites including lipids and sterols need to be tightly regulated in order to match the cellular demand. In particular, cholesterol, the most abundant individual lipid, has profound influence on membrane properties. Thus, cholesterol levels are modulated by uptake, endogenous synthesis, intracellular storage as cholesteryl ester, and efflux to apolipoproteins that altogether maintain membrane cholesterol homeostasis and prevent deleterious effects of hypercholesterolemia. Cholesterol is synthesized in the endoplasmic reticulum (ER) by the mevalonate pathway in a multistep process (1). One of the intermediates of cholesterol biosynthesis, farnesyl pyrophosphate (FPP), serves also as the building block of several additional molecules including dolichol and ubiquinone, and it is utilized in posttranslational modifications such as glycosylation and prenylation (2). Since these isoprenoids affect important biological activities, commitment to the nonsterol branch of the mevalonate pathway needs to be tightly controlled. On one side, enzymes of the mevalonate pathway are transcriptionally upregulated by the sterol regulatory element binding protein (SREBP) in response to low cholesterol (3). On the other hand, rate-limiting mevalonate pathway enzymes, HMG-CoA reductase (HMGCR) and squalene epoxidase (SQLE), are under posttranslational feedback inhibition (4, 5, 6, 7). When there is no demand for synthesis, the abundance of these key enzymes is downregulated by the ER-associated degradation (ERAD) pathway, a mechanism whereby proteins are targeted for turnover by the proteasome (8). While these homeostatic mechanisms determine the overall biosynthesis rate, SQLE has been suggested to influence the flux between the sterol and nonsterol arm of the mevalonate pathway (5, 6, 7) (Fig. S1*A*). However, a truncated SQLE form (SQLE-S) that persist cholesterol-induced degradation has been shown recently (9), posing the question of how pathway selection is solely accommodated at SQLE step. Underlying most of these homeostatic responses to fluctuating amounts of cholesterol is the sensing of its level by a number of regulatory ER membrane proteins *via* conserved sterol-sensing domains (SSDs) (10). At low cholesterol level, SSD-mediated protein-protein interaction between Insig and Scap is destabilized enabling trafficking of SREBP from the ER to the Golgi, where the transcription factor domain is released and activated by regulated proteolysis by site-1 and site-2 proteases (11). Conversely, under conditions of high cholesterol, HMGCR is targeted to a redundant set of ERAD E3 ubiquitin ligases, leading to its extraction from the membrane and proteasomal turnover (7, 12, 13, 14, 15). In addition to these multiple ERAD dislocation mechanisms by the E3 ubiquitin ligases, cells have a noncanonical proteolytic arm of the ERAD pathway that is centered around signal peptide peptidase (SPP) (16). SPP is an aspartic intramembrane protease related to Alzheimer’s disease-associated γ-secretase (17). In addition to its role in processing signal peptides, SPP was shown to be also involved in protein degradation, functionally interacting with the ERAD E3 ubiquitin ligases TRC8 and MARCH6 (18, 19, 20, 21, 22). After these fundamental findings, further impact of SPP-triggered degradation on cellular physiology and the mechanism of its regulation remain as interesting research questions. In this study, we show that SPP and TRC8 trigger cholesterol-dependent proteasomal degradation of squalene synthase (SQS; gene name, *FDFT1*), an enzyme acting at the junction of sterol and nonsterol arms, thereby serving as a metabolic control at this branching point of the mevalonate pathway. We hypothesize that this mechanism potentially contributes to pathway selection between the two branches, as it was also suggested for SQLE (4, 5, 6, 7).

## Results

### Substrate proteomics identifies SQS as a physiological SPP substrate

**Figure 1:**
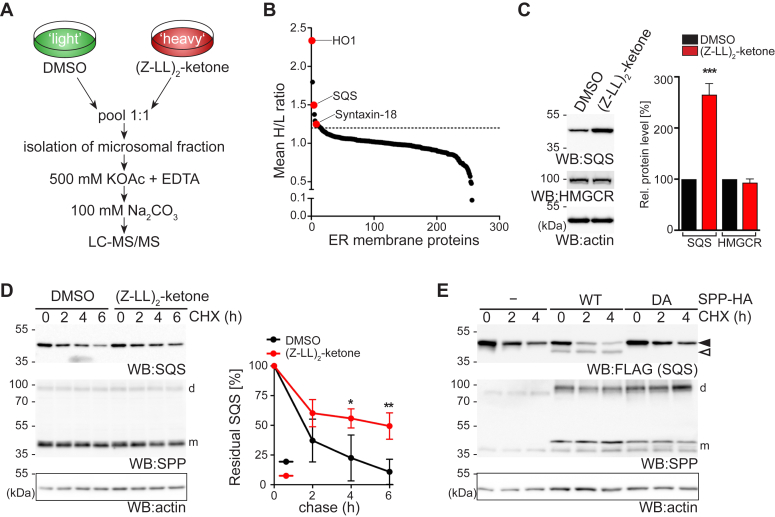
**Differential organelle proteomics identifies SQS as an SPP substrate.***A*, experimental outline of the SILAC-based organelle proteomics. *B*, ER-resident membrane proteins quantified by organelle proteomics (mean H/L ratio, *n* = 2). *C*, Western blot (WB) showing steady state analysis of endogenous SQS and HMGCR in Hek293T cells upon 16 h treatment with (Z-LL)_2_-ketone or DMSO. Actin was used as loading control. Quantification shows Mean ± SD, *n* = 4. *D*, cycloheximide (CHX) chase of endogenous SQS in Hek293T cells in presence of (Z-LL)_2_-ketone or vehicle control (DMSO). Monomeric and SDS-stable dimeric forms of SPP are indicated (m and d, respectively). Actin was used as loading control. Mean ± SD, *n = 3*. *E*, CHX chase of ectopically expressed FLAG-tagged SQS in Hek293T cells co-expressing HA-tagged wt SPP and catalytic mutant SPP^D265A^ (DA) revealing processing of the full-length form (*black arrow*) to an N-terminal fragment (*white arrow*). See also Fig. S1. DMSO, dimethyl sulfoxide; HA, hemagglutinin; HMGCR, HMG-CoA reductase; SPP, signal peptide peptidase; SQS, squalene synthase.

To identify endogenous substrates for SPP, we compared the proteome of ER-derived microsomes from Hek293T cells, with or without treatment with the SPP inhibitor (Z-LL)_2_-ketone (17), using stable-isotope labeling by amino acids in cell culture and quantitative proteomics (Fig. 1*A*). We identified 15 ER-resident proteins that showed an increased steady-state level of more than 20% in the inhibitor treated condition (see File S1). In addition to the previously identified SPP substrates heme oxygenase 1 (HO1) and syntaxin-18 (19, 22, 23), inhibition of SPP led to an increased level of SQS (Fig. 1*B*), an ER-resident tail-anchored protein that defines the critical branching point of the sterol arm of the mevalonate pathway, by converting FPP into squalene (Fig. S1*A*) (1). In contrast, 69 additional tail-anchored proteins identified were not significantly changed in their abundance (File S1), indicating that SPP is highly specific. Consistent with an SPP-mediated degradation of SQS, Western blot analysis of (Z-LL)_2_-ketone treatment showed a significant increase of its steady-state levels, whereas HMGCR levels remained unchanged (Fig. 1*C*). Consistent increase of SQS levels was observed also in HeLa and U2OS cells (Fig. S1*B*). Furthermore, inhibition of SPP delayed SQS turnover in a cycloheximide chase assay (Fig. 1*D*). We observed generation of a (Z-LL)_2_-ketone-sensitive endogenous SQS cleavage fragment migrating just below the full-length form upon SPP WT overexpression (Fig. S1*C*). In agreement with this, coexpression of a FLAG-tagged SQS construct with SPP destabilized the full-length form and a faster migrating band was observed, whereas ectopic expression of the catalytically inactive mutant SPP^D265A^ had no effect (Fig. 1*E*). The tail-anchored protein Ubc6 was completely resistant to SPP-catalyzed cleavage (Fig. S1*D*), supporting the notion that SPP is highly specific.

A key determinant of SPP substrates are helix-destabilizing residues in the N-terminal portion of the transmembrane (TM) domain, as well as specific amino acid residues surrounding the cleavage site (24). Mutation of either of two conserved TM serine residues, namely S388 and S397 (Fig. S1*E*), which match the SPP substrate consensus, completely stabilized SQS in the ER (Fig. S1, *F* and *G*). This result indicates that a membrane-integral degradation signal (degron) determines recognition of SQS by SPP. In order to confirm such a direct substrate-enzyme interaction, we performed coimmunoprecipitation experiments with the dominant-negative SPP^D265A^ mutant (18, 23), revealing efficient trapping of endogenous SQS, whereas the ER protein CLIMP63 did not interact (Fig. S1*H*). Taken together, these results show that SQS is a substrate for the SPP-dependent noncanonical ERAD pathway.

### SQS is targeted for degradation by Hrd1 as well the SPP-TRC8-dependent ERAD pathway

**Figure 2:**
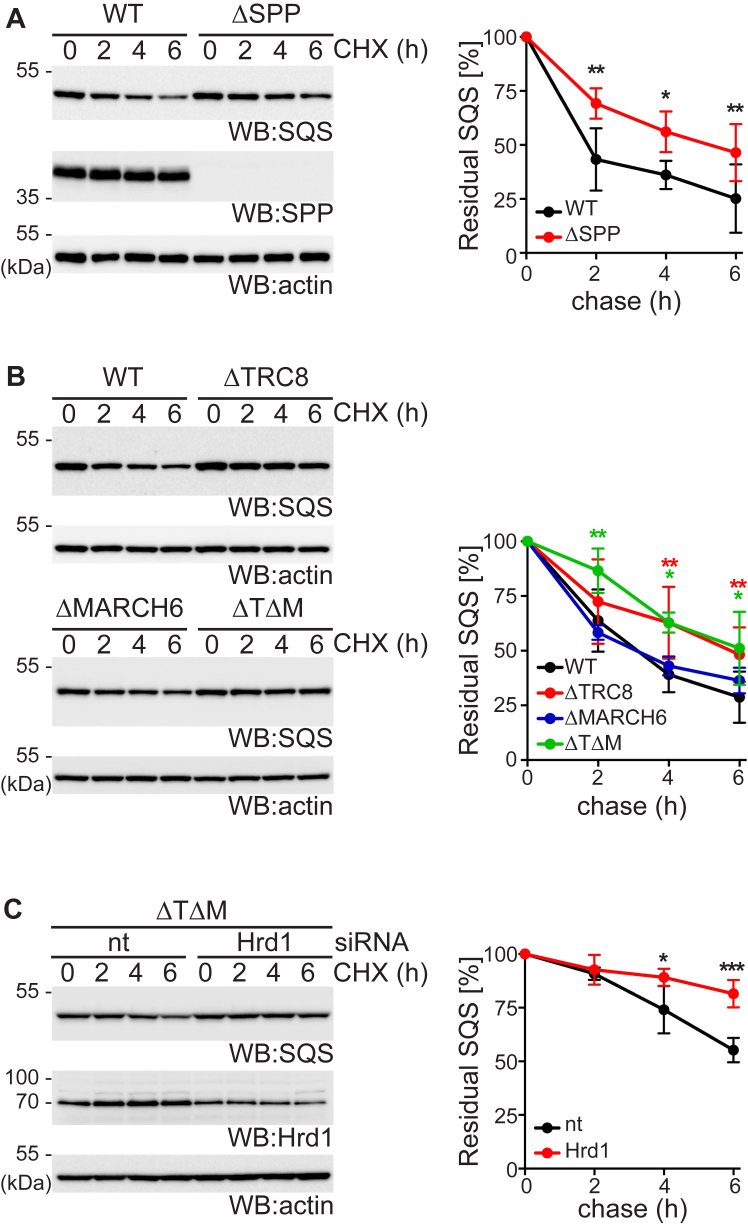
**SPP and TRC8 define a noncanonical ERAD pathway that acts on SQS in parallel to Hrd1 pathway.***A*, stability of endogenous SQS in Hek293T wt and Hek293TΔSPP cells assessed by cycloheximide (CHX) chase assay and analyzed by Western blot (WB) analysis. Equal protein loading was controlled by actin on a separate WB. Mean ± SD, *n = 3*. *B*, SQS degradation assay in Hek293T wt, ΔTRC8, ΔMARCH6, and ΔTRC8ΔMARCH6 (ΔTΔM) double-deficient Hek293T cells. Mean ± SD, *n = 3*. *C*, SQS degradation assay in ΔTΔM cells transfected with nontargeting (nt) or Hrd1 siRNA. Mean ± SD, *n = 3*. See also Fig. S2. ERAD, ER-associated degradation; SPP, signal peptide peptidase; SQS, squalene synthase.

Previous studies showed that membrane insertion of SQS depends on the ER membrane protein complex and defective insertion upon its loss leads to decreased stability of SQS, which was suggested to be independent of canonical ERAD pathways at this condition (25, 26). In order to investigate how SQS turnover relates to the SPP-specific ERAD machinery, we generated SPP KO Hek293T cells. Although non-conditional SPP KO mice die after embryonic day 13.5 (27), SPP-deficient tissue culture cells grow normal, while accumulating the unprocessed substrate HO1 and show a defect in processing of the hepatitis C virus core protein (Fig. S2, *A* and *B*) (19, 27). Consistent with our proteomics analysis, we observed significant steady-state increase of endogenous SQS, but not HMGCR in Hek293T SPP KO cells (Fig. S2*C*), whereas SQS mRNA level was not affected (Fig. S2*D*). Although there was still substantial turnover of SQS in SPP KO cells, cycloheximide chase assay showed a significant increase of the half-life compared to the WT cells (Fig. 2*A*). This result indicates that, SQS is subject to more than one ERAD pathway as had been observed for HMGCR (Fig. S1*A*). We next generated KO cells of both E3 ubiquitin ligases that had been linked to SPP, namely TRC8 and MARCH6 (18, 20, 21), and analyzed SQS protein stability and mRNA expression levels (Figs. 2*B* and S2*E*). Similar to SPP KO, we observed a partial stabilization of SQS in ΔTRC8 cells, whereas MARCH6 KO did not show any significant effect (Figs. 2*B* and S2*F*). Combined KO of TRC8 and MARCH6 did not further stabilize SQS (Fig. 2*B*). On the other hand, knockdown of the Hrd1 E3 ubiquitin ligase by siRNA transfection also interfered with SQS degradation (Fig. S2*G*). Strikingly, Hrd1 knockdown in the TRC8 and MARCH6 double-deficient cells further stabilized SQS (Fig. 2*C*). Consistent with SPP and TRC8 acting in one linear ERAD pathway, addition of the SPP inhibitor (Z-LL)_2_-ketone did not show any effect in ΔSPP or ΔTRC8 cells but stabilized SQS in ΔMARCH6 cells (Fig. S2, *H*–*J*). Taken together, these results show that SQS is subject to at least two independent branches of the ERAD pathway, the Hrd1-dependent dislocation route and the SPP-TRC8 pathway (18, 19).

### High cholesterol accelerates SPP-TRC8 mediated SQS degradation

**Figure 3:**
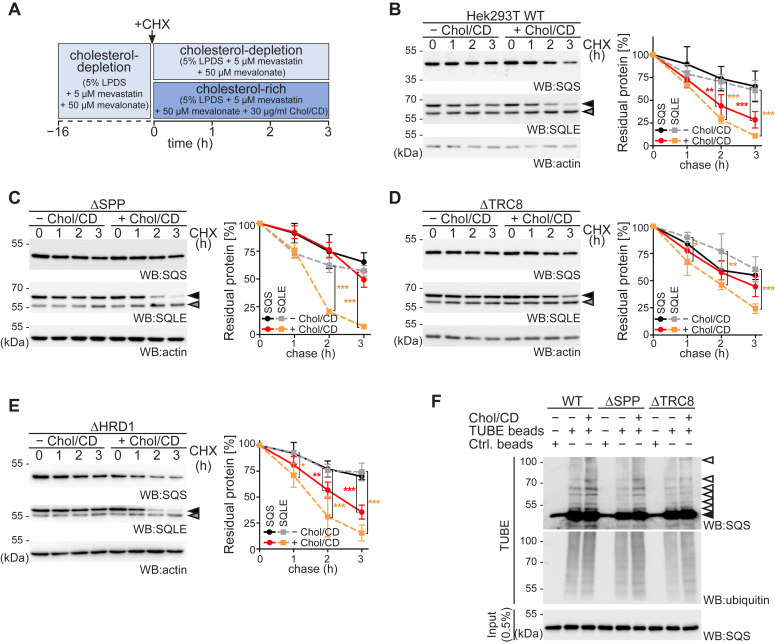
**Cholesterol activates SPP-TRC8 complex for regulatory degradation of SQS.***A*, experimental outline of the sterol starvation and cholesterol repletion assay. *B*, degradation of endogenous SQS and the full-length form of SQLE (*black arrow*) were assayed in Hek293T wt cells treated as outlined in (*A*) and analyzed by Western blot (WB) analysis. Actin was used as loading control for SQS; signal for SQLE was obtained from a separate WB. *Gray arrow*, cholesterol-insensitive truncated SQLE-S. Mean ± SD, *n = 3*. *C*, SQS and SQLE degradation assay in Hek293TΔSPP cells as in (*B*). *D*, SQS and SQLE degradation assay in Hek293TΔTRC8 cells as in (*B*). *E*, SQS and SQLE degradation assay in Hek293TΔHRD1 cells as in (*B*). *F*, Hek293T wt, ΔSPP, and ΔTRC8 cells were grown in sterol-depletion medium and treated with epoxomicin and incubated in cholesterol depletion or repletion as outlined in (*A*) before cell lysates were subjected to affinity enrichment using TUBE2 agarose beads revealing ubiquitinated species (*white arrows*). Control (Ctrl.) agarose beads were used to confirm TUBE2 specificity. See also Fig. S3. Chol/CD, cholesterol complexed to methyl-β-cyclodextrin; LPDS, lipoprotein-depleted serum; SPP, signal peptide peptidase; SQLE, squalene epoxidase; SQS, squalene synthase.

To assess whether SPP-TRC8-mediated SQS turnover is regulated, we studied its degradation rate in response to cholesterol levels. For this, we used lipoprotein-free medium and conditions blocking endogenous cholesterol synthesis, while synthesis of essential non-sterol isoprenoids is permitted and no toxicity was observed (Figs. 3*A* and S3*A*) (28, 29). SQS was significantly more stable in Hek293T cells grown in cholesterol-depletion medium when compared to cholesterol replete conditions (Fig. 3*B*). The effect observed was comparable to cholesterol-induced ERAD of full-length SQLE (Fig. 3*B*) (7), whereas the truncated SQLE-S form was stable, as observed before (9). In contrast, in SPP- or TRC8-deficient cells, SQS turnover was delayed and completely uncoupled from cholesterol levels, whereas cholesterol-induced ERAD of SQLE was unaffected by both KOs (Fig. 3, *C* and *D*). This effect was specific to cholesterol, since 25-hydroxycholesterol, which serves as a potent trigger of HMGCR turnover (30), and epi-cholesterol had milder effects on SQS stability (Fig. S3*B*), as well as on SQLE stability as previously had been observed (5). In Hrd1 deficient cells, on the other hand, cholesterol addition still led to accelerated degradation of SQS, similar to what we observed in WT cells (Fig. 3*E*). Taken together, our results show that for both enzymes a basal turnover exists. At high cholesterol conditions, SQLE is degraded by a MARCH6- and p97-dependent dislocation route (7), and SQS becomes subject to SPP-TRC8-triggered turnover. Consistent with a cholesterol-induced ubiquitination of SQS, we observed increased levels of several higher molecular weight SQS species in a ubiquitin-specific pulldown in WT cells (Fig. 3*F*). This difference was diminished in ΔTRC8 cells but still observed in the absence of SPP (Fig. 3*F*). These results suggest that, TRC8-catalyzed ubiquitination serves as the sterol-sensitive step that is succeeded by SPP-catalyzed release from the ER membrane.

### TRC8 triggers SPP-catalyzed SQS cleavage

**Figure 4:**
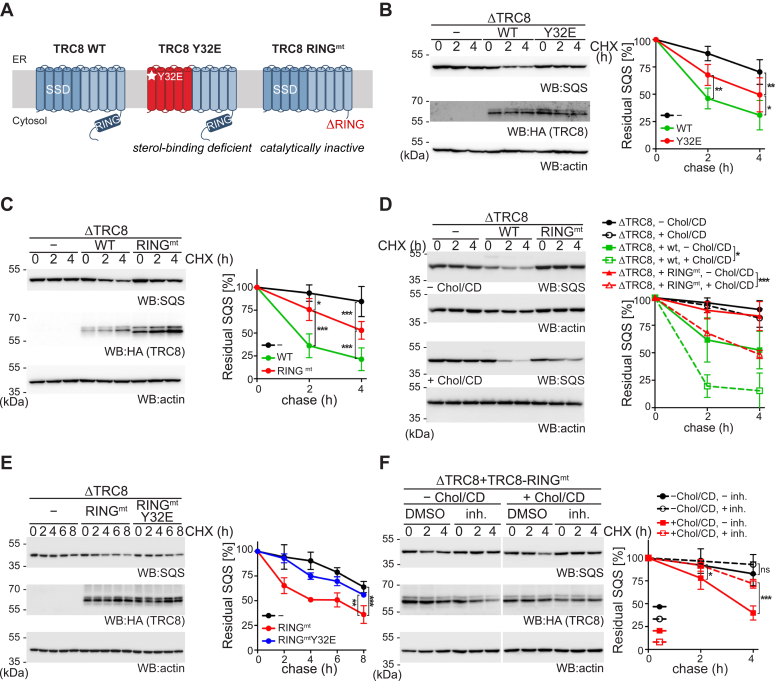
**TRC8 acts as an allosteric activator of SPP-catalyzed SQS cleavage.***A*, outline of TRC8 constructs used. *B*, doxycycline-induced expression of HA-tagged TRC8 wt or the Y32E mutant in Hek293ΔTRC8 cells. Cells were subjected to CHX chase and assayed for endogenous SQS. Actin was used as loading control. Mean ± SD, *n = 3*. *C*, doxycycline-induced expression of HA-tagged TRC8 wt or RING domain mutant (RING^mt^) in Hek293ΔTRC8 cells. Cells were subjected to CHX chase and assayed for endogenous SQS. Mean ± SD, *n = 3*. *D*, doxycycline-induced expression of HA-tagged TRC8 wt or the RING^mt^ in Hek293ΔTRC8 cells in cholesterol depletion or repletion. Cells were assayed for endogenous SQS by CHX chase as outlined in Figure 3*A*. The actin control for + Chol/CD samples was obtained from a separate WB. Mean ± SD, *n = 3*. *E*, doxycycline-induced expression of HA-tagged TRC8 RING^mt^ or the RING^mt^ Y32E double mutant in Hek293ΔTRC8 cells. Cells were subjected to CHX chase assay and assayed for endogenous SQS. Mean ± SD, *n = 3*. *F*, doxycycline-induced expression of HA-tagged TRC8-RING^mt^ in Hek293ΔTRC8 cells was assayed for endogenous SQS by CHX chase in presence or absence of (Z-LL)_2_-ketone (inh.). Actin was used as loading control for TRC8; signal for SQS was obtained from a separate WB. Mean ± SD, *n = 4*. HA, hemagglutinin; SPP, signal peptide peptidase; SQS, squalene synthase; WB, Western blot.

Previous studies showed that TRC8 levels are modulated by sterols; decreased levels upon sterol rich conditions, and it interacts with sterol biogenesis regulating proteins Insig, SREBP2 and Scap (31, 32). It was also shown to affect the processing and stability of SREBP precursor proteins and was suggested to fine tune SREBP response upon prolonged sterol depletion (31, 32). Therefore, we analyzed the potential sensor role of TRC8 on sterol regulated degradation of SQS. Expression of TRC8 harboring a tyrosine-32-glutamate (Y32E) mutation, which previously has been suggested to compromise its SSD (32), showed significantly reduced activity in triggering SQS degradation in ΔTRC8 cells compared to the WT construct (Fig. 4, *A* and *B*). Interestingly, deletion of TRC8’s catalytic RING domain (RING^mt^) only partially abrogated rescue of the KO phenotype (Fig. 4, *A* and *C*), indicating that TRC8 has also a noncatalytic function in SQS turnover by sensing the cholesterol level. Consistent with this hypothesis, the noncatalytic function of TRC8-RING^mt^ was entirely cholesterol dependent (Fig. 4*D*) and the RING^mt^-Y32E double mutant of TRC8 did not rescue SQS degradation in ΔTRC8 cells (Fig. 4*E*). In addition, SPP inhibition under high cholesterol conditions in ΔTRC8 cells expressing TRC8-RING^mt^ led to a significant stabilization of SQS level (Fig. 4*F*), suggesting an allosteric activation of SPP proteolytic activity mediated through TRC8. Altogether, these results indicate that high cholesterol conditions enhance the proteolytic processing of SQS by SPP. Moreover, for complete SQS turnover, both the E3 ubiquitin ligase and the sterol sensing-activity of TRC8 are required.

### SPP-mediated SQS degradation has impacts on cholesterol levels

**Figure 5:**
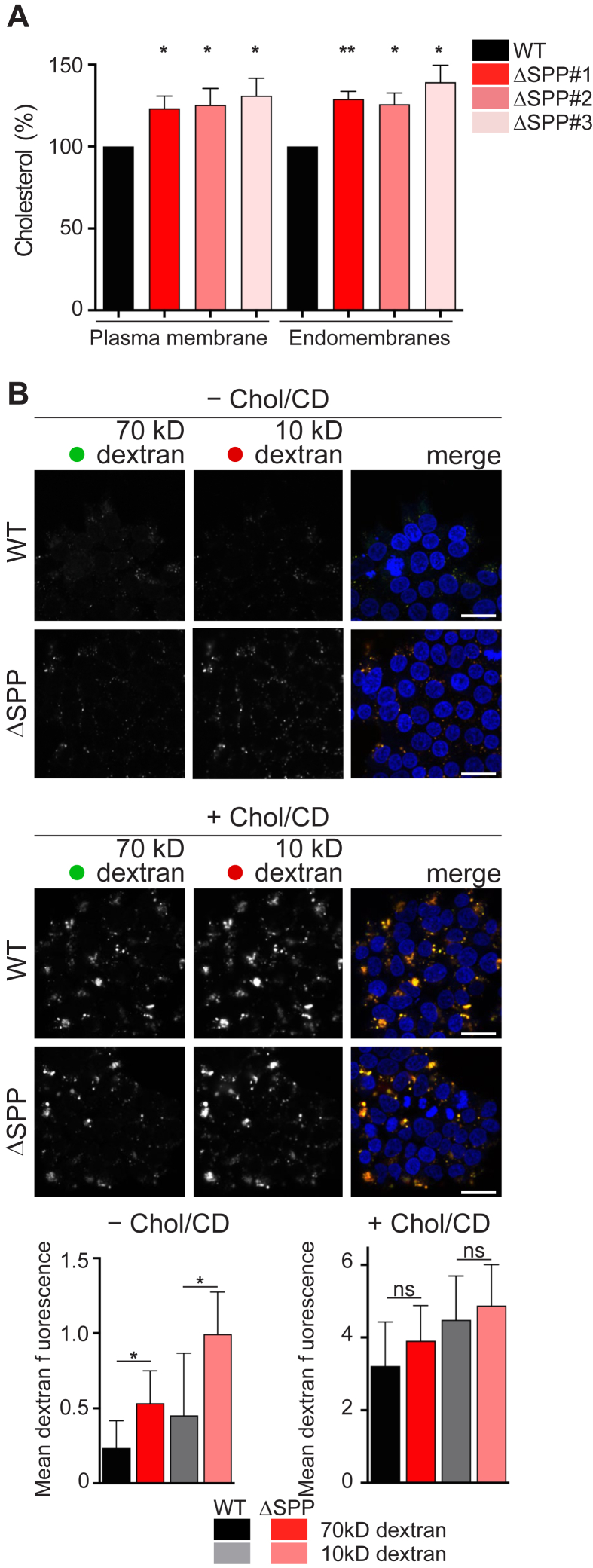
**SPP-mediated SQS quantity regulation plays a crucial role in the control of cholesterol homeostasis.***A*, lipidomic analysis of subcellular fractionation samples comparing cholesterol levels in Hek293T wt and three ΔSPP cell lines (clone #1, #2, #3). Mean ± SD, *n* = *3*. *B*, Hek293T wt and ΔSPP cells were grown in sterol-depletion medium (*top panel*) or in cholesterol-rich medium (*bottom panel*) and incubated with fluorescently labeled 70-kDa dextran (*green*) and 10-kDa dextran (*red*). Intracellular dextran was quantified after 30 min uptake. Nuclear DNA was stained by Hoechst 33342 (*blue*). Mean ± SD, *n* = 10 fields of view. The scale bar represents 100 μm. See also Figs. S4 and S5. SPP, signal peptide peptidase; SQS, squalene synthase.

Next, we tested the influence of SPP mediated SQS abundance control on cellular cholesterol levels by analyzing the lipidome of WT and ΔSPP Hek293T cells (Fig. S4, *A–E*). In order to uncouple cellular cholesterol level from receptor-mediated uptake (1), cells were grown in lipid-depleted medium. Total cholesterol levels were significantly increased in all three ΔSPP clones (Fig. S5*A*). The difference was even more pronounced in the lipidomic analysis upon subcellular fractionation, showing a striking increase of cholesterol in both endomembrane and plasma membrane fractions (Fig. 5*A*). No difference in the levels and processing of the SREBP2 transcription factor were observed under the same lipid-deficient conditions for lipidomic analysis (Fig. S5*B*), indicating that the effect is due to shunting the metabolite FPP into the cholesterol-branch of the mevalonate pathway and is not a consequence of an overall activation of lipid synthesis (Fig. S1*A*).

The levels of cholesterol in the cell needs to be tightly controlled and even small changes can have profound effects. As a way to test how ablation of SPP affects plasma membrane properties and to characterize the KO phenotype, we measured uptake of fluorescently labeled dextran from the medium. Consistent with cholesterol-dependent endocytosis (33), in Hek293T cells grown in rich medium 70-kDa dextran was visible in various vesicles, whereas no internalization was observed in cholesterol-depleted medium (Fig. 5*B*). Strikingly, significantly more 70-kDa dextran was internalized in ΔSPP cells, compared to WT, in cholesterol depletion conditions (Fig. 5*B*). Increase of the endocytosis rate in ΔSPP cells was even more pronounced for the uptake of 10-kDa dextran (Fig. 5*B*), which, in addition to macropinocytosis, also becomes subject to a less-well-defined pinocytosis route (33). While the detailed molecular mechanism of the altered endocytosis rate needs further analysis, our results are in line with a prominent role of the SPP-mediated SQS abundance control mechanism in fine-tuning cellular cholesterol levels. Total lipidome analysis of Hek293T wt and ΔSPP cells did not show any major differences in glycerophospholipid and storage lipid content. However, we observed an overall reduction in sphingolipid levels in ΔSPP cells (Fig. S5*C*), as well as an increase in long chain phosphatidylserine (PS) (36:1, Fig. S4*C*) that has been linked to positioning of cholesterol in the plasma membrane (34). The mechanism of how SPP influences sphingolipid homeostasis and coupling of cholesterol to membrane composition remain important questions for future studies.

## Discussion

**Figure 6:**
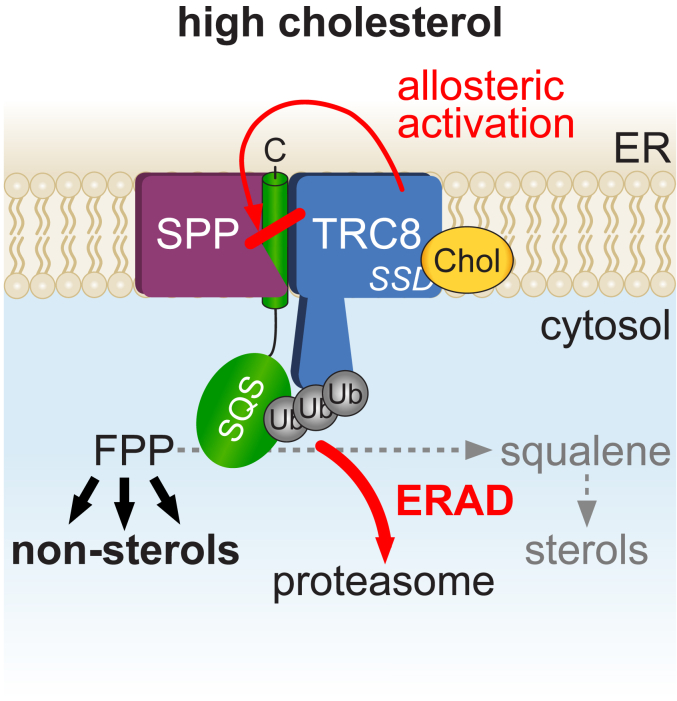
**Model for SPP-triggered cholesterol dependent degradation of SQS.** At high levels, cholesterol is sensed by the sterol-sensing domain (SSD) of TRC8 in Hek293T wt cells leading to ubiquitination and SPP-mediated ERAD-R (regulatory ERAD) of SQS. We emphasize that the order of events remains speculative. ERAD, ER-associated degradation; SPP, signal peptide peptidase; SQS, squalene synthase.

Regulation of metabolite levels according to cellular needs is crucial to maintain homeostasis. Here we show that the regulatory arm of the ERAD pathway (ERAD-R) centered around SPP (16) acts as a so far unrecognized metabolic regulation in the mevalonate pathway (Fig. 6). Since the SQS-catalyzed conversion of two molecules of FPP to squalene determines to which extent the mevalonate pathway generates sterol or other biologically important nonsterol molecules including dolichol and ubiquinone, we hypothesize that it potentially serves as a metabolic switch at the branching point of the mevalonate pathway (Fig. 6). This regulated degradation can act as a fast-reacting fine-tuning process to control the flux of the FPP intermediate into two arms of the mevalonate pathway according to the cellular needs. While HMGCR, as the first rate-limiting enzyme of the multistep pathway, determines the overall flux of acetyl-CoA into isoprenoid biosynthesis, regulated turnover of SQS defines a control step that shunts FPP between the sterol and nonsterol branches. Cholesterol levels are increased in SPP KO cells, due to increased SQS abundance. This shows that, in addition to the control of the rate-limiting enzyme HMGCR by multiple ERAD E3 ubiquitin ligases (7, 12, 13, 14), cholesterol also acts as a negative feedback regulator on the branching-point enzyme SQS. Consistent with this, we observed that the ERAD E3 ubiquitin ligases, TRC8 and Hrd1, show an overlapping substrate spectrum, potentially allowing integration of different signals to control the abundance of SQS and ensuring plasticity of the response. How the complex cellular metabolism of nonsterol mevalonate products influences this control and whether there are compensating mechanisms that suppress an SPP KO phenotype, as well as how an SPP upregulation observed in cancer cells (35, 36) impacts this mechanism, remain to be interesting questions for future research.

Our findings suggest that SPP is activated partially by a noncatalytic sterol-sensing function of TRC8 to cleave SQS. This observation shed light to a long-standing question about the physiological relevance of the predicted SSD in TRC8 (32). As noted previously, TRC8 levels are also regulated by sterol amounts in the cell, by being downregulated at sterol rich conditions (31, 32). We believe TRC8 mediated degradation of SQS is part of a complex multilevel metabolic regulation, acting as distinct control steps during the time course of cellular response to sterol depletion and repletion. Different to ERAD of HMGCR (4) and activation of the transcription factor SREBP (11), 25-hydroxycholesterol does not show a pronounced effect on the SPP-TRC8-mediated protein abundance control of SQS. This difference indicates that, for this ERAD-R mechanism the end product of cholesterol biosynthesis is sensed and not the general flux through the mevalonate pathway is monitored. The molecular mechanism of how TRC8’s SSD activates ERAD-R remains to be determined. However, we assume that recognition of single-pass TM substrates by the SPP-TRC8 complex is independent of Scap/Insig adaptor proteins that are known to control HMGCR and SREBP (37). Since SPP specificity is determined by TM substrate features (24), we suggest that the cholesterol-induced increase of the SQS processing rate is caused by allosteric activation of SPP by the membrane-integral SSD of TRC8. Presenilin, a mechanistically related aspartic intramembrane protease serving as the active subunit of the γ-secretase complex, is also modulated by cholesterol, which impacts generation of neurotoxic amyloid-β peptides in Alzheimer’s disease (38). A recent γ-secretase structure revealed three distinct cholesterol-binding sites in the noncatalytic subunit Aph-1 adjacent to presenilin (39), suggesting that allosteric regulation of aspartic intramembrane proteases by lipids is a more general principle. Overall, intramembrane proteolysis emerges as a fast and irreversible posttranslational modification that controls the activity and abundance of various organellar membrane proteins (16).

Human cancer association studies showed that SPP expression is increased in glioblastoma, lung, and breast cancer cells (35, 36). Based on our findings, we hypothesize that reduced SQS level upon increased SPP expression and concomitant increased flux of FPP into dolichol and ubiquinone synthesis may help to handle the increased metabolic load of rapidly growing cancer cells. Consistent with this idea, a recent study showed that synthesis through the mevalonate pathway is shifted toward ubiquinone to sustain viability of p53-deficient cancer cells exposed to metabolic stress (40). Likewise, heme metabolism may contribute to settle the microenvironment for cancer cell growth. These new insights in the role of SPP in cellular metabolism control offer the prospect of novel therapeutic intervention strategies.

## Experimental procedures

### Antibodies

The following antibodies were used in this study: rabbit polyclonal anti-SPP (kind gift of C. Schaller), mouse monoclonal anti-FLAG (M2, Sigma-Aldrich, #F1804), rat monoclonal anti-HA (3F10, Sigma-Aldrich, #11867423001), mouse monoclonal anti-CLIMP63 (G1/296, Enzo Life Sciences, #ENZ-ABS669-0100), mouse monoclonal anti-β actin (AC-15, Sigma-Aldrich, #A1978), mouse monoclonal anti-ubiquitin (P4D1, Santa Cruz Biotechnology, #sc-8017), rabbit polyclonal anti-HO1 (Enzo Life Sciences, #ADI-SPA-896-F), rabbit polyclonal anti-SQLE (Proteintech, #12544-1-AP), rabbit monoclonal anti-SQS (Abcam, #ab109723), rabbit monoclonal anti-SQS (Abcam, #ab195046), mouse monoclonal anti-GFP (Sigma-Aldrich, #11814460001), mouse monoclonal anti-HMGCR (Merck, #MABS1233), rabbit polyclonal anti-Synoviolin (Hrd1) (Bethyl Laboratories, #A302-946A), rabbit polyclonal anti-Calnexin (Abcam,#ab22595), goat polyclonal anti-SREBP2 (Novus Biologicals, #AF7119), AlexaFluor488 goat anti-mouse IgG (H + L) (Invitrogen, #A-11029).

### Plasmids

Plasmids based on pcDNA3.1 (Invitrogen) encoding human SPP with a triple hemagglutinin-tag inserted between residue 373 and the C-terminal KKEK ER-retention signal (SPP-HA) was described previously (18). The active-site mutant SPP D265A was introduced by site-directed mutagenesis (Stratagene). Human SQS (*FDFT1*, Gene ID 2222, full-ORF Gateway complementary DNA clone 101218403) and Ubc6-FLAG (Gene ID 51465, IMAGE 8322482) with an N-terminal triple FLAG-tag were inserted as *EcoR*I/*Xho*I-fragment into pcDNA3.1. TM mutants of SQS were generated by site-directed mutagenesis. For the generation of doxycycline-inducible cell lines, we introduced constructs for human TRC8 (Gene ID 11236), TRC8-RING^mt^ lacking residues 547 to 580 (gifts from R. Gemill (41)), and the Y32E SSD mutant generated by site-directed mutagenesis (this study), with a C-terminal single hemagglutinin-tag respectively, into pcDNA5/FRT/TO (Invitrogen). Plasmids encoding an ER-targeting signal sequence fused to a red fluorescent protein (RFP) followed by a KDEL ER-retention signal (RFP-KDEL) (42) and the N-terminal 195 residues of the hepatitis C virus polyprotein (Glasgow strain genotype 1a) corresponding to the core protein and four amino acids of the envelope protein E1 fused to an N-terminal triple FLAG-tag (43) have been described before.

### Cell culture and transfection

Hek293T (American Type Culture Collection [ATTC]), Hek293 Flp-In T-Rex (Thermo Fisher Scientific), HeLa (ATTC) and U2OS (Gift from B. Dobberstein) cells were maintained without antibiotics in Dulbecco’s modified Eagle’s medium (DMEM) (Invitrogen) supplemented with 10% fetal bovine serum (FBS) (Invitrogen) and grown as monolayers at 37 °C and 5% CO_2_. Cell lines were routinely tested for *mycoplasma* throughout this study and were free from *mycoplasma* contamination. Hek293 Flp-In T-REx cells with inducible expression of the protein of interest were additionally supplemented with 100 μg/ml hygromycin B (Invitrogen) and 10 μg/ml blasticidin (Gibco). Transient transfections were performed using 40-kDa linear polyethyleneimine (PEI MAX, Polysciences) (44). Typically, 1 μg plasmid encoding the substrate and 500 ng of plasmid encoding wt or mutant SPP were used per well of a 6-well plate. Total DNA (2 μg/well) was held constant by adding empty plasmid. If not stated otherwise, cells were harvested 24 h after transfection. For inhibition of the proteasome and SPP, 2 μM epoxomicin and 50 μM (Z-LL)_2_-ketone (Merck Millipore) were added from stock solutions in dimethyl sulfoxide (DMSO). As vehicle control, the same amount of DMSO was used. For the induction of HO1, 20 μM hemin (ferriprotoporphyrin IX chloride, Sigma-Aldrich) was added to the cells from a 20 mM stock solution in 0.05 M NaOH and incubated overnight. For immunofluorescence staining, Hek293T cells were seeded onto 12 mm cover slips in 24-well plates and transfected with 500 ng plasmid encoding substrate and 150 ng RFP-KDEL. Total amount of DNA (1 μg/well) was held constant with empty plasmid.

### CRISPR/Cas9-mediated gene editing

CRISPR single guide RNA (sgRNA) oligonucleotide sequences targeting SPP (*HM13* gene), TRC8, or MARCH6 were designed using the E-CRISP online tool (45). The sgRNAs were cloned into the *Bbs*I-digested p x 459 (pSpCas9(BB))-2A-Puro V2.0, (Addgene #62988) vector, verified by sequencing and used for transient transfection of Hek293T cells and Hek293 Flp-In T-REx cells. sgRNA-targeted cell populations were selected with 3 μg/ml puromycin, and clonal cell lines were generated by limited dilution. KO clones for SPP were identified by immunoblotting and validated by sequencing of genomic DNA. Validation of TRC8 and MARCH6 KO cells was based on indel detection. Identification of indels was performed using the online tools TIDE (46), CRISP-ID (47), and ICE v2 (48) and shown in Fig. S6. The following sgRNAs were used: SPP-1 (clone #1 and #3) GCCCTCAGCGATCCGCATAA, SPP-2 (clone #2) GCCTGAAACAATCACCAGCC, TRC8 CCAGACATACTACGAGTCTT, MARCH6 TATCATCCTTGTGTATGTAC. The Hrd1 KO cells were described previously (49).

### Generation of stable Hek293 Flp-In T-REx cell lines

For the generation of doxycycline-inducible stable cell lines, Flp-In T-REx Hek293ΔTRC8 cells were cotransfected with pOG44 (Invitrogen) and either pcDNA5/FRT/TO/TRC8, pcDNA5/FRT/TO/TRC8-RING^mt^, or pcDNA5/FRT/TO/TRC8-Y32E and cells were selected with hygromycin B (100 μg/ml, Invitrogen).

### siRNA knockdown of Hrd1

For the knockdown of Hrd1 (*SYVN1* gene), cells were transfected with an ON-TARGETplus siRNA smart pool (L-007090-00-0005) obtained from Dharmacon. As control, nontargeting siRNA pool (D-001810-01) was used. Cells were seeded onto poly-L-lysine (PLL)-coated 12-well plates in DMEM supplemented with 10% FBS and transfected using the RNAiMAX reagent (Life Technologies) according to the manufacturer’s instructions. After 24 h, medium was exchanged to fresh medium. Cells were harvested 48 h after transfection. The final molarity of siRNA per well was 20 picomol.

### Mass spectrometry-based substrate screen

For the identification of SPP substrates, we used stable isotope labeling by amino acids in cell culture-based quantitative organelle proteomics. For this, Hek293T cells were grown for five doublings in medium supplemented with either heavy amino acids (^13^C_6_^15^N_4_-L-Arg and ^13^C_6_^15^N_2_-L-Lys; Silantes) or unlabeled amino acids before applying (Z-LL)_2_-ketone (50 μM) or DMSO as vehicle control. For each condition ((Z-LL)_2_-ketone or DMSO), 3 × 150 mm plates were used.

If not stated otherwise, all steps were performed at 4 °C. At day of harvest, cells were washed with ice-cold 1x PBS (10 mM NaH_2_PO_4_, 1.4 mM KH_2_PO_4_, 2.7 mM KCl, 140 mM NaCl, pH 7.4), harvested in PBS-EDTA (1x PBS, 1 mM EDTA, pH 8.0, 0.2 g/l glucose) and an equal number of cells were resuspended in ice-cold hypotonic buffer (10 mM Hepes-KOH, pH 7.4, 1.5 mM MgCl_2_, 10 mM KOAc, 0.5 mM DTT) containing 10 μg/ml PMSF and Complete EDTA-free protease cocktail (Roche). Cells were incubated on ice for 10 min and lysed by passing five times through a 27-Gauge needle, followed by centrifugation for 10 min at 400*g* to remove cell debris and nuclei. The supernatant was laid on top of a low salt sucrose cushion (500 mM sucrose, 50 mM Hepes-KOH, pH 7.4, 50 mM KOAc, and 5 mM Mg(OAc)_2_) and centrifuged for 20 min at 100,000*g* in a 70Ti rotor (Beckman Coulter). The resulting membrane pellet was resuspended in 1 ml rough microsome (RM) buffer (250 mM sucrose, 50 mM Hepes-KOH, pH 7.4, 50 mM KOAc, 2 mM Mg(OAc)_2_, 1 mM DTT) and extracted by 500 mM KOAc and 50 mM EDTA on ice for 15 min, followed by centrifugation through a high salt sucrose cushion (500 mM sucrose, 50 mM Hepes-KOH, pH 7.4, 500 mM KOAc, 5 mM Mg(OAc)_2_) for 20 min at 100,000*g*. The pellet thereof was resuspended in 1 ml freshly prepared, ice-cold sodium carbonate (100 mM) buffer and transferred onto an alkaline sucrose cushion (125 mM sucrose, 100 mM Na_2_CO_3_) followed by centrifugation for 20 min at 100,000*g*. The pellet was resuspended in RM buffer and snap frozen in liquid nitrogen.

For proteomics, pellets in RM buffer were diluted in 2x SDS-sample buffer (see below) separated by SDS-PAGE for 1 cm, and samples were excised from the gel before reducing with DTT and alkylated with iodoacetamide. Digestion with trypsin was done overnight at 37 °C, followed by quenching with 0.1% TFA (Biosolve), and the supernatant was dried in a vacuum concentrator before LC-MS analysis. Nanoflow LC-MS^2^ analysis was performed using an Ultimate 3000 LC system coupled to an QExactive HF mass spectrometer (Thermo Fisher Scientific). Samples were dissolved in 0.1% TFA and loaded onto a C18 Acclaim PepMap100 trap-column (Thermo Fisher Scientific) with a flow rate of 30 μl/min 0.1% TFA. Subsequently, peptides were eluted and separated on an C18 Acclaim PepMap RSLC analytical column (75 μm × 250 mm, Thermo Fisher Scientific) with a flow rate of 300 nl/min in a 120 min gradient of 3% buffer A (0.1% formic acid) to 40% buffer B (0.1% formic acid/acetonitrile). The mass spectrometer was operated in data-dependent acquisition mode, automatically switching between MS and MS^2^. Collision induced dissociation MS^2^ spectra were generated for up to 20 precursors with normalized collision energy of 29%.

Processing of RAW files was performed using MaxQuant (v. 1.5.3.30, https://www.maxquant.org/). MS^2^ spectra were searched against the Uniprot human proteome database and the contaminants database by Andromeda search engine with the following parameters: Carbamidomethylation of cysteine residues, acetylation of protein N termini, and oxidation of Met were considered as variable modifications. Trypsin as the proteolytic enzyme with up to two missed cleavages was allowed. The maximum false discovery rate was 0.01 and a minimum peptide length of seven amino acids was required. All other parameters were default parameters of MaxQuant. Quantitative normalized ratios were calculated by MaxQuant and used for further data analysis.

### Lipidomics

In order to determine the change in lipid composition, Hek293T wt cells together with three different Hek293TΔSPP clones were analyzed by lipidomics. At day 0, cells were seeded onto PLL-coated 100 mm dishes in DMEM supplemented with 10% FBS. At day 1, cells were first washed two times with 1x PBS followed by sterol-depletion in medium containing DMEM supplemented with 5% lipoprotein deficient serum (LPDS) (see below). At day of harvest (day 2 after seeding), membrane fractions were prepared by subcellular fractionation (see below). The last centrifugation step was performed for 60 min at 21,000*g* at 4 °C in a table-top centrifuge. Membranes were resuspended in methanol and subjected to lipid extractions using an acidic Bligh & Dyer, except from plasmalogens, which were extracted under neutral conditions as described in (50). Lipid standards were added prior to extractions, using a master mix containing phosphatidylcholine (13:0/13:0, 14:0/14:0, 20:0/20:0; 21:0/21:0; Avanti Polar Lipids), sphingomyelin (d18:1 with N-acylated 13:0, 17:0, 25:0, semi-synthesized (50)), D_6_-cholesterol (Cambridge Isotope Laboratories), phosphatidylinositol (16:0/16:0 and 17:0/20:4; Avanti Polar Lipids), phosphatidylethanolamine (PE), PS and phosphatidylglycerol (14:1/14:1, 20:1/20:1, 22:1/22:1, semi-synthesized (50), diacylglycerol (DAG, 17:0/17:0, Larodan), cholesterol ester (9:0, 19:0, 24:1, Sigma-Aldrich), triacylglycerol (TAG, D_5_-mix, LM-6000/D_5_-17:0,17:1,17:1; Avanti Polar Lipids), ceramide (Cer) and glucosylceramide (HexCer) (both d18:1 with N-acylated 15:0, 17:0, 25:0, semi-synthesized (50), lactosylceramide (LacCer, d18:1 with N-acylated C12 fatty acid; Avanti Polar Lipids), phosphatidic acid (17:0/20:4; Avanti Polar Lipids), and lyso-phosphatidylcholine (17:1; Avanti Polar Lipids). PE plasmalogen (PE-P)-containing standard mix was supplemented with PE P-mix 1 (16:0p/15:0, 16:0p/19:0, 16:0p/25:0), PE P-mix 2 (18:0p/15:0, 18:0p/19:0, 18:0p/25:0), and PE P-Mix 3 (18:1p/15:0, 18:1p/19:0, 18:1p/25:0). Semisynthesis of PE-P- was performed as described in (51). Lipid extracts were resuspended in 60 μl methanol and samples were analyzed on an AB SCIEX QTRAP 6500+ mass spectrometer (SCIEX) with chip-based (HD-D ESI Chip; Advion Biosciences) nano-electrospray infusion and ionization *via* a Triversa Nanomate (Advion Biosciences) as previously described (51). Resuspended lipid extracts were diluted 1:10 in 96-well plates (Eppendorf twin tec 96, colorless, Z651400-25A; Sigma-Aldrich) prior to measurement. Lipid classes were analyzed in positive ion mode applying either specific precursor ion (PC, lyso-PC, sphingomyelin, cholesterol, Cer, HexCer, Hex2Cer, and PE-P) or neutral loss (PE, PS, phosphatidylinositol, phosphatidylglycerol, and phosphatidic acid) scanning as described in (51). Data evaluation was performed using LipidView (RRID: SCR_017003; SCIEX, https://sciex.com/products/software/lipidview-software) and an in-house-developed software (ShinyLipids).

### Quantitative real-time PCR

Quantitative real-time PCR was used to assess the SQS mRNA level (transcribed from the *FDFT1* gene) in Hek293T wt cells or cells deficient for SPP, TRC8, MARCH6, or TRC8/MARCH6-double deficient cells. Therefore, RNA was isolated from confluent cells from one well of a 6-well plate with the NucleoSpin RNA isolation kit (Macherey-Nagel) according to manufacturer’s protocol. A total of 2 μg RNA were reverse transcribed using the RevertAid First Strand cDNA Synthesis Kit (Thermo Fisher Scientific). Quantitative PCR was performed using the SensiFAST SYBR No-ROX kit (Bioline) according to the manufacturer’s protocol and the LightCycler480 Instrument II (Roche) with the following cycle settings: preincubation for 2 min at 95 °C, followed by 40 cycles amplification at 95 °C for 5 s, 57 °C for 10 s, and 72 °C for 25 s. Each reaction was performed in technical triplicate. TATAA-box binding protein and β-2 microglobulin were used as reference genes. Primers used for TATAA-box binding protein: 5′-CCGGCTGTTTAACTTCGTT and 5′-ACGCCAAGAAACAGTGATGC; β-2 microglobulin: 5′-CACGTCATCCAGCAGAGAAT and 5′-TGCTGCTTACATGTCTCGAT; SQS/*FDFT1*: 5′-GAGGACTTCCCAACGATCTCC and 5′-AACTCTGCCATCCCAATGCC. Relative quantification of gene expression was calculated as described previously (52).

### Preparation of LPDS, cholesterol inclusion complexes, and cholesterol-depletion and repletion assay

For the manipulation of the cellular cholesterol content, Hek293T cells were pretreated and treated with DMEM containing 5% LPDS. LPDS was prepared from FBS by density gradient ultracentrifugation using solid potassium bromide according to (53) with minor modifications. The density adjusted FBS was subjected to ultracentrifugation for 25 h at 310,500*g* at 10 °C in a 70Ti rotor (Beckman Coulter) before dialysis. The dialyzed fraction was sterile filtered and frozen at −20 °C.

Cholesterol repletion was carried out by incubating cells in the presence of cholesterol/methyl-β-cyclodextrin inclusion complexes (Chol/CD) which were prepared with minor modifications according to (54). Cholesterol (Sigma-Aldrich) was dissolved in isopropanol:chloroform (2:1) and added in small aliquots to a stirring 5% (w/v) methyl-β-cyclodextrin (Sigma-Aldrich) solution in a water bath heated to 80 °C. The solution was cooled, sterile filtered, and kept at 4 °C protected from light until use. The cholesterol to methyl-β-cyclodextrin molar ratio was 10:1.

For experiments involving sterol depletion and repletion, cells were seeded onto PLL-coated plates. After 24 h, cells were washed two times with 1x PBS before switching to sterol-depletion medium containing 5% LPDS, supplemented with 5 μM mevastatin and 50 μM mevalonate (Sigma-Aldrich). After overnight sterol depletion in this medium, cells were treated with fresh sterol-depletion medium either in absence or in presence of 30 μg/ml Chol/CD complexes or other test agents.

### Crystal violet cytotoxicity assay

For crystal violet cell viability assay, cells were seeded onto PLL-coated 24-well plates, sterol-depleted overnight with medium containing LPDS, mevastatin, and mevalonate and treated with or without 30 μg/ml Chol/CD. At 3 h after treatment, cells were washed with 1x PBS and fixed for 10 min at 4 °C with cold methanol/acetone (1:1; 1 ml/well). Cells were stained with 0.1% crystal violet in 25% methanol for 15 min followed by washing four times with tap water. Afterward, the stained cells were air dried overnight and representative images were acquired using a Zeiss Axio Observer Z1 widefield microscope equipped with a LD-Plan-NEOFLUAR 20x/0.4 Air Ph2 objective at 20x magnification. For quantitative measurement, adsorbed dye was solubilized with methanol containing 0.1% SDS by gentle agitation for 2 h. Absorbance at 570 nm (*A*_570_) from three biological replicates and each replicate in technical duplicate was measured using the Infinite M1000 microplate reader (Tecan Trading AG) and accompanying software i-control (v.1.10.4.0). For quantification, the mean absorbance for each cell line and condition tested was corrected by subtracting the mean *A*_570_ of blank wells. The *A*_570_ of FBS-treated cells for each cell line was set to 1.0 and the respective fold-change under treatment conditions for each cell line was calculated.

### Cycloheximide chase experiments

Cycloheximide (100 μg/ml) chase was performed 24 h after transfection of Hek293T cells or after doxycycline induction of Hek293 Flp-In T-REx cells. For chase of endogenous proteins, cycloheximide was added 48 h after seeding of Hek293T cells. Following the addition of the compound in medium onto cells, lysates were prepared at indicated time points and protein abundance was analyzed by Western blotting. Time point 0 was immediately harvested and did not receive cycloheximide.

### Subcellular fractionation

In order to isolate cellular membranes, subcellular fractionation was performed. Therefore, cells from 6-well or 100 mm dishes were washed with 1x PBS followed by adding 1 ml PBS-EDTA to detach cells. Cells were centrifuged for 10 min at 400*g* at 4 °C, the supernatant was removed, and the cell pellet was resuspended in 1 ml hypotonic buffer supplemented with 10 μg/ml PMSF and protease inhibitor cocktail (Roche). Cells were incubated on ice for 10 min, lysed by passing 5 times through a 27-Gauge needle, followed by centrifugation for 5 min at 1000*g* at 4 °C to pellet cells and debris. To pellet membranes, the postnuclear supernatant was centrifuged for 30 min at 100,000*g* at 4 °C using a S120AT2 rotor (Beckman Coulter). The resulting pellet containing the membranes was resuspended in SDS sample buffer (see below).

Fractionation into plasma membrane and the ER-containing endomembrane fraction for the lipidome analysis was performed using the Minute Plasma Membrane Protein Isolation and Cell Fractionation Kit (Invent Biotechnologies, Inc) according to manufacturer’s instructions.

### Fluorescence microscopy and fluid phase uptake assay

For immunofluorescence staining, cells were first carefully washed with 1x PBS followed by fixation with 0.5 ml/well 4% formaldehyde in PBS for 20 min with gentle agitation. Afterward, cells were washed three times with 1x PBS and then permeabilized with 0.2 ml/well 0.5% Triton X-100 in PBS for 10 min at 4 °C. Following washing with 1x PBS (three times), unspecific binding sites were blocked by incubating the cells with blocking buffer (20% FBS in 1x PBS) for 30 min. Primary antibody in blocking buffer supplemented with 0.01% Triton X-100 was applied for 1 h with gentle agitation followed by washing three times with 1x PBS, 5 min each. After this, the secondary antibody at respective dilution in blocking buffer was applied onto the cells and incubated for 1 h with agitation. From this step onward, cells were protected from light to prevent antibody bleaching. Cells were washed three times with 1x PBS, 5 min each and mounted in Fluoromount-G (SouthernBiotech). Confocal analysis was performed on a Zeiss LSM 780 inverted microscope. Lasers used were a 458 nm, 488 nm, and 514 nm Argon laser (25 mW) and a 561 nm diode-pumped solid-state laser (20 mW). Images were acquired using a Plan-APOCHROMAT 63x/1.4 oil objective at 1024 × 1024 pixels with line average 4 and a pinhole size of 1 Airy unit using the ZEN 2012 (v. 1.1.2.0, https://www.zeiss.com/microscopy/en/products/software/zeiss-zen.html) software. For image stacks, series of 8 to 12 sections with 0.5 μm along the Z-axis were taken. Images were processed and maximum intensity projections created using Fiji.

In order to assess the influence of cholesterol on dextran internalization, fluid phase uptake assays were done. For this, cells were kept overnight in sterol-depleted medium (see above). The next day, cells were either left under sterol depletion or in repletion medium with cholesterol for 3 h. For the last 30 min, cells were incubated with 10 kDa dextran-Alexa568 and 70 kDa dextran-Oregon Green 488 at 0.1 mg/ml each. Subsequently, cells were washed two times with ice cold PBS and fixed with cold 4% formaldehyde in PBS for 15 min. After fixation, cells were washed in PBS, stained with 5 μg/ml Hoechst 33342 stain in PBS for 5 min, and finally rinsed with PBS prior to microscopy. Cellular uptake of fluorescently labeled dextran was analyzed by confocal microscopy using a Zeiss LSM 780 inverted microscope and quantified using Fiji/ImageJ (http://rsb.info.nih.gov/ij/) according to (55).

### Immunoprecipitation and TUBE-ubiquitin pull-down

For immunoprecipitation, cells were washed once with 1x PBS followed by treatment with the membrane-permeable cross-linker dithiobis succinimidyl propionate (2 mM) in PBS for 30 min on ice. Tris–HCl, pH 7.4 was added to a final concentration of 20 mM for 15 min to quench the cross-linking reaction. Cells were washed with 1x PBS, harvested in PBS-EDTA followed by centrifugation for 3 min at 900*g* at 4 °C. The supernatant was discarded, and the pellet was lysed in 1 ml solubilization buffer (50 mM Hepes-KOH, pH 7.4, 150 mM NaCl, 1.5 mM MgCl_2_, 1 mM EGTA, and 10% glycerol) containing 10 μg/ml PMSF, complete protease inhibitor cocktail and 1% Chaps. Cells were lysed for 30 min on ice followed by centrifugation for 10 min at 13,000 rpm at 4 °C. The supernatant was diluted 1:1 with solubilization buffer without detergent and lysates were precleared by incubation with Protein G sepharose beads (GE HealthCare) on a rotation wheel for 2 h, at 4 °C. For immunoprecipitation, precleared lysates were spun for 3 min at 4000*g* at 4 °C, transferred to a clean tube and incubated with monoclonal anti-HA agarose beads (Sigma-Aldrich) for 4 h, at 4 °C. Beads were washed three times with wash buffer (50 mM Hepes–KOH, pH 7.4, 150 mM NaCl, 1.5 mM MgCl_2_, 1 mM EGTA, 10% glycerol) containing 0.1% Chaps and eluted in 2x SDS-sample buffer (see below).

For determining the ubiquitination of endogenous SQS, Hek293T, Hek293TΔSPP, and Hek293TΔTRC8 cells were seeded onto PLL-coated 100 mm cell culture dishes. Cells were washed two times with 1x PBS and treated overnight with medium containing 5% (v/v) LPDS. Prior to harvest, the proteasome inhibitor epoxomicin (final 5 μM) was added to each dish and one dish of each cell line received 30 μg/ml Chol/CD for 3 h. Cells were washed once with 1x PBS and harvested in 1x PBS-EDTA supplemented with 20 mM *N*-ethylmaleimide to block deubiquitinating enzymes followed by centrifugation for 3 min at 900*g* at 4 °C. Cells were lysed for 20 min on ice in 500 μl solubilization buffer containing freshly added protease inhibitor cocktail, PMSF, 1% Triton X-100 and 20 mM *N*-ethylmaleimide. Lysates were clarified by centrifugation for 15 min at full speed at 4 °C, and the supernatant was diluted 1:1 with solubilization buffer without detergent. Lysates were precleared at 4 °C for 1 h on a rotating wheel using 30 μl Protein-S agarose beads (Merck Millipore) slurry. Beads were centrifuged for 3 min at 4000*g* and the precleared lysate transferred to a fresh tube. Affinity purification/enrichment of ubiquitinated species was performed using 20 μl TUBE2 agarose bead (LifeSensors Inc) slurry, incubating overnight at 4 °C on a rotating wheel. Beads were washed three times with TBS-T (50 mM Tris–HCl, pH 7.4, 150 mM NaCl, and 0.1% Tween-20). Proteins were eluted by heating to 65 °C for 15 min in 20 μl 1x SDS-sample buffer. Samples were subjected to SDS-PAGE and Western blotting (see below).

### SDS-PAGE and Western blotting

Proteins were solubilized in SDS sample buffer (50 mM Tris–HCl, pH 6.8, 10 mM EDTA, pH 8.0, 5% glycerol, 2% SDS, and 0.01% bromophenol blue) containing 5% β-mercaptoethanol. Samples were heated to 65 °C for 15 min with agitation, except for immunoblots of Hrd1 and HMGCR which were incubated at 37 °C for 30 min. For Western blotting, proteins were separated by reducing Tris-glycine SDS-PAGE and transferred to polyvinylidene difluoride (Merck Millipore) membranes followed by blocking, antibody incubation, and enhanced chemiluminescence analysis (Advansta) using the LAS4000 (Fuji) or ImageQuant800 (Cytiva) systems. For reprobing, polyvinylidene difluoride membranes were incubated in glycine stripping buffer (100 mM glycine, 20 mM Mg(OAc)_2_, 50 mM KCl, 1% Tween-20, 2.5% SDS), pH 2.2, for 20 min followed by washing 6 times for 10 min with TBS-T before processing as outlined above.

### Statistical analysis

Quantification of Western blots was performed using Fiji ImageJ (https://imagej.net/software/fiji/). Statistical analysis was performed using GraphPad Prism v.5.00 (GraphPad Software, Inc, https://www.graphpad.com/features). Differences between two means in steady state analysis was determined by two-tailed unpaired Student’s *t* test. The difference between multiple means in cycloheximide chase assays was determined by two-way ANOVA test followed by Bonferroni post hoc test. If not stated otherwise, data are represented as mean ± SD and result from at least three independent biological replicates. Significance levels were as follows: ∗*p* ≤ 0.05, ∗∗*p* < 0.01, ∗∗∗*p* < 0.001.

## Data availability

Proteomics data is included in the supporting information. Raw lipidomic data file can be shared upon request. Other data are contained within the manuscript.

## Supporting information

This article contains supporting information (1, 5, 6, 7, 27, 56, 57).

## Conflict of interest

The authors declare that they have no conflicts of interest with the contents of this article.
